# The involvement of transient receptor potential channels in mast cell activation by microbubbles

**DOI:** 10.1111/jcmm.17947

**Published:** 2023-09-07

**Authors:** Jia Liu, Long Qing, Yufei He, Qihui Zhu, Weigang Xu, Jianhua Wu

**Affiliations:** ^1^ Department of Dermatology, Changhai Hospital Second Military Medical University (The First Affiliated Hospital of Naval Medical University) Shanghai China; ^2^ Department of Naval Diving Medicine, Naval Medical Center Naval Medical University Shanghai China; ^3^ Department of Diving and Hyperbaric Medical Research, Naval Medical Center Naval Medical University Shanghai China

**Keywords:** calcium, decompression sickness, degranulation, mast cell, microbubble, transient receptor potential

## Abstract

This study was to explore the activation of mast cells by microbubbles, with the focus on transient receptor potential (TRP) channels mediated degranulation and calcium influx. Bone marrow‐derived mast cells (BMMCs) were primarily obtained from femurs in mice and induced differentiation for 4 weeks. After the purity identification, BMMCs were contacted by homogeneous microbubbles with the diameter of 1 mm for 1 h. β‐hexosaminidase and histamine levels in supernatants were assessed by enzyme‐linked immunosorbent assay (ELISA) and the CD63 expression was tested by flow cytometry. The intracellular calcium binding with Fluo‐4 AM dyes in BMMCs was observed under the fluorescence microscope and the mean fluorescence intensity was quantitatively measured by flow cytometry. β‐hexosaminidase release, histamine concentration, CD63 expression and calcium influx were significantly increased in BMMCs group upon microbubble stimulation compared to the control groups. After preconditioning with the available inhibitors and microbubble contact, only transient receptor potential vanilloid 1 (TRPV1) and TRPV4 inhibitors robustly suppressed the microbubble‐induced degranulation. Likewise, the elevated fluorescence intensity of cytosolic calcium level was also significantly weaken. The results demonstrated microbubble stimulus effectively promoted BMMCs degranulation, which could be substantially restrained by inhibitors targeted for blocking TRPV1 or TRPV4 channel. The alternation of intracellular calcium level in BMMCs was consistent with the changes of degranulation capacity. It's suggested that the activation of BMMCs by microbubbles may involve specific TRP calcium dependent channels.

## 1 INTRODUCTION

Skin lesion is one of the most common symptoms of decompression sickness (DCS), which may occur in divers, pilots and astronauts by decompressing improperly from higher atmospheric pressure and the desaturated bubbles might play an important part.^1^, ^2^ Cutis marmorata and livedo reticularis are the characteristic manifestations of skin lesions, which is often regarded as a clinical sign of severe DCS.^3^ So far, the mechanism of DCS skin lesions has not been fully elucidated, and the hypothesis of autochthonous bubbles have been widely accepted as the primary mechanism, still there is lack of sufficient experimental evidence. Available research results show significant inflammatory reactions occurring in skin lesions, including neutrophil infiltration, endothelial dysfunction, inflammatory molecules release in circulation and vascular permeability alternation. It was speculated that subcutaneous bubbles arising from rapid decompression maybe the key factor which lead to the cascade reactions within skin tissues.^4^ If the autochthonous bubble hypothesis is the case, how does it work?

Mast cell is an important element in innate immunity and crucial effector cell in modulation of immune responses and regulation of inflammatory reactions.^5^ Skin‐resident mast cells fight against the external environmental allergens or pathogens and play a vital role in allergic skin disorders.^6^ Upon activation, mast cells could release a broad array of preformed granule‐stored mediators, including histamine, serotonin and newly generated pro‐inflammatory mediators such as cytokines and chemokines.^7^ These biological substances subsequently trigger the inflammation cascade reactions and allergic responses.^8^ A significant increase amount of mast cells can be seen in the histopathology of skin tissues, which implied the functional role of mast cells on the aetiopathogenesis of several cutaneous diseases.^9^ The underlying mechanism of DCS skin lesions probably lies in the interplay between decompression microbubbles and mast cells activity.

Mechanical stimuli activated mast cells have been demonstrated in several studies, such as the needle acupuncture, high temperature during moxibustion and red laser light applied in acupoints area for analgesia therapy. Mast cells degranulation accompanied with the intracellular free calcium rise and ATP release following the physical stimulation.^10^ Experiments conducted on vascular endothelial cells and neurons have thrown light on the mechanical‐ biological signal transduction in bubble‐cell contact depended on the TRP channels activation. TRP channels with subgroups such as the canonical (TRPC), vanilloid (TRPV), melastatin (TRPM), ankyrin (TRPA), polycystin (TRPP) and mucolipin (TRPML) are widely expressed on mast cell membranes and discovered to be sensitive to temperature, voltage, tension and other physical stimuli, which contributes to calcium influx and regulate biological activity.^11^ Hence it's postulated that microbubbles acted as an effective irritate to provoke mast cell activation and increase degranulation via TRP‐mediated signal pathway.

## 2 MATERIALS AND METHODS

### 2.1 Animals

C57BL/6 male mice aged 6–8 weeks with the weight of 18 ~ 22 g were obtained from the Jeisijie Experimental Animal Co.Ltd. All the mice were kept under specific pathogen‐free condition in accordance with the guidelines of internationally accepted humane standards. The experimental protocols were carried out with the approval of the Institutional Animal Care and Use Ethics Committee of Naval Medical University.

### 2.2 Culture of BMMCs

The primary culture of bone marrow‐derived mast cells (BMMCs) was originally isolated from the murine bilateral femur. Briefly, after euthanized by cervical dislocation, bone marrow cavity of mouse femur was repeatedly washed with Roswell Park Memorial Institute medium 1640 (RPMI1640, Gibco) to collect the rinsing fluid. Next the suspension was filtered through 100 μm cell strainer and centrifuged the filtrate, the cells were resuspended in RPMI1640 medium, supplemented with 10% fetal bovine serum (FBS, Gibco), 10 ng/mL recombinant murine IL‐3 (rmIL‐3, PeproTech), 10 ng/mL recombinant murine stem cell factor (rmSCF, PeproTech) and 1% antibiotics & antimycotics (penicillin & streptomycin & amphotericinB, Gibco). Cells were kept at a density of 1 × 10^6^ cells/mL and inoculated into culture flasks for 4 weeks.

### 2.3 Identification of BMMCs

At the fourth week, BMMCs were resuspended and washed in phosphate‐ buffered saline (PBS) with a concentration of 5 × 10^5^ cells/mL, and cell suspensions were smeared onto the glass slides followed by toluidine blue staining for 5 min. Mixed with equal volume deionized water for 15 min. Subsequently, cells were indirectly rinsed with deionized water twice and tapped dry. The coverslip was placed over the stained cells. Images were observed and photographed at 200× magnification.

The surface membrane expressions of both CD117 and FcεRIα were quantitatively assessed by flow cytometry. Cells were resuspended and washed twice in PBS followed by centrifugation. Single‐cell suspensions were stained with the FITC‐labelled FcεRIα monoclonal antibody (ThermoFisher) or/and PE‐labelled CD117 monoclonal antibody (ThermoFisher) respectively and incubated in the dark for 30 min. After labelling, cells were washed and suspended in PBS. The fluorescently labelled samples were measured on CytoFLEX BD Immuno‐cytometry Systems and the data were analysed using CytExpert software.

When both the glass slides showed typical morphology of BMMCs (BMMCs showed suspension state in growth, round and similar in shape, and the nuclei observed blue and purple heterochromatic particles scattered in the cytoplasm after toluidine blue staining) and the FcεRIα^+^ and CD117^+^ expressed above 90%, the cells were used for subsequent experiments (Figure S1).

### 2.4 Microbubbles stimulation to BMMCs

**FIGURE 1:**
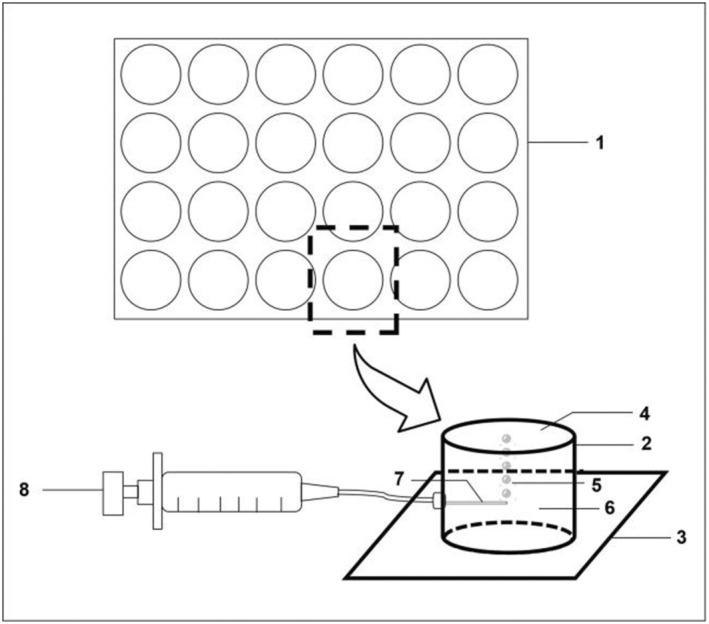
Diagram of microbubbles contacting with BMMCs. (1) 24‐well plate, (2) part of a plate, (3) coverslip, (4). BMMCs, (5) microbubbles, (6) RPMI1640 containing 20% FBS, (7) microneedle, (8) syringe. BMMCs, bone marrow‐derived mast cells; RPMI, park memorial institute medium; FBS, fetal bovine serum.

The standardized BMMCs were resuspended in RPMI1640 (1 × 10^6^ cells/mL) and transferred in 24‐well plates with 1 mL volume per well. After centrifugation at 300× g for 10 min at 4°C, bubble medium (RPMI1640 containing 20% FBS) was gently add to each well until the maximum capacity. Covered the coverslips and then inverted the plate. By virtue of liquid surface tension, the medium in the wells could not flow out. In the experimental group, the microneedle was inserted into well via the reserved micropore and a monolayer of microbubbles with a diameter of 1 mm was formed by slowly pushing the syringes (Figure 1). Through the buoyancy force, the microbubbles stayed upper to contact the BMMCs gathered in the bottom of the wells for 1 h. This practical device was authorized by the Patent Office of China (Patent Number: ZL 2022 21198753.4). In the control group without microbubbles contact at different time‐points examined, the plates were turned over and directly remove the medium after centrifuged.

### 2.5 TRP inhibitors pretreatment

For inhibitor intervention experiments, a batch of cells were respectively pre‐incubated with HC030031 (TRPA1 inhibitor, 10 μM), SKF96365 (TRPC inhibitor, 20 μM), HC067047 (TRPV4 inhibitor, 1 μM), SB705498 (TRPV1 inhibitor, 20 μM), 9‐phenanthrol (TRPM4 inhibitor, 20 μM), AMG333 (TPRM8 inhibitor, 100 μM), ML‐SI3 (TPRML inhibitor, 20 μM) or EIPA (TRPP3 inhibitor, 100 μM). All the inhibitors were purchased from MedChemExpress and incubated for 1 h at 4°C with the vehicle control (with DMSO). After washing with PBS twice, all the groups were intervened by microbubble stimulus for 1 h as described above.

All the experimental groups were stopped after centrifugation, the supernatants were collected to evaluate the degranulation capacity with enzyme‐linked immunosorbent assay (ELISA) and the cell pellets were resuspended with PBS and then with fluorescence staining for flow cytometry analysis.

### 2.6 Determination of BMMCs degranulation and activation

BMMCs were centrifuged in all groups after treatment. Both the secretion level of β‐hexosaminidase and histamine in supernatants, were served as the main indicators of mast cell degranulation, and assessed by ELISA kit (ELK Biotechnology & NJJC Bioengineering).

The surface activated marker CD63 of mast cell was detected and quantified by flow cytometry. With the density of 1 × 10^6^ cells/mL, 2 μL anti‐mouse APC‐labelled CD63 antibodies (BioLegend) were applied in 400 μL volume. After incubation in the dark for 30 min, cells were washed and suspended in PBS. The percentage of CD63 positive mast cells and mean fluorescence intensity were measured on BD flow cytometer and data were analysed using CytExpert software.

### 2.7 Measurement of intracellular calcium

BMMCs were loaded by a fluorescent dye Fluo‐4 AM (MedChemExpress) with excitation at 494 nm and emission at 516 nm. In brief, cells were added with Fluo‐4 AM (1 mM stock) to a final concentration of 5 μM, and incubated at 37°C for 30 min in dark. After washing and resuspending with PBS, the cells were incubated for another 30 min at 37°C. Subsequently, cells were preconditioned with different inhibitors. For the microbubbles stimulation, cells were intervened by the method described above. Finally, marked cell nuclei with DAPI staining dye (Vectorlabs) and collected the stained cells. The representative images of the intracellular calcium were taken by fluorescent microscopy (DMi8, Leica) and processed by ImageJ software. Simultaneously the mean fluorescence intensities were measured by flow cytometry.

### 2.8 Statistical analysis

The data was expressed as means ± standard deviation (SD). One‐way anova was performed for comparison among multiple groups. Then SNK test was used for pairwise comparison and Dunnett‐*t* test was used to compare multiple experimental groups with the control group. *p* < 0.05 was accepted as statistically significant.

## 3 RESULTS

### 3.1 BMMCs degranulation and activation by microbubbles

**FIGURE 2:**
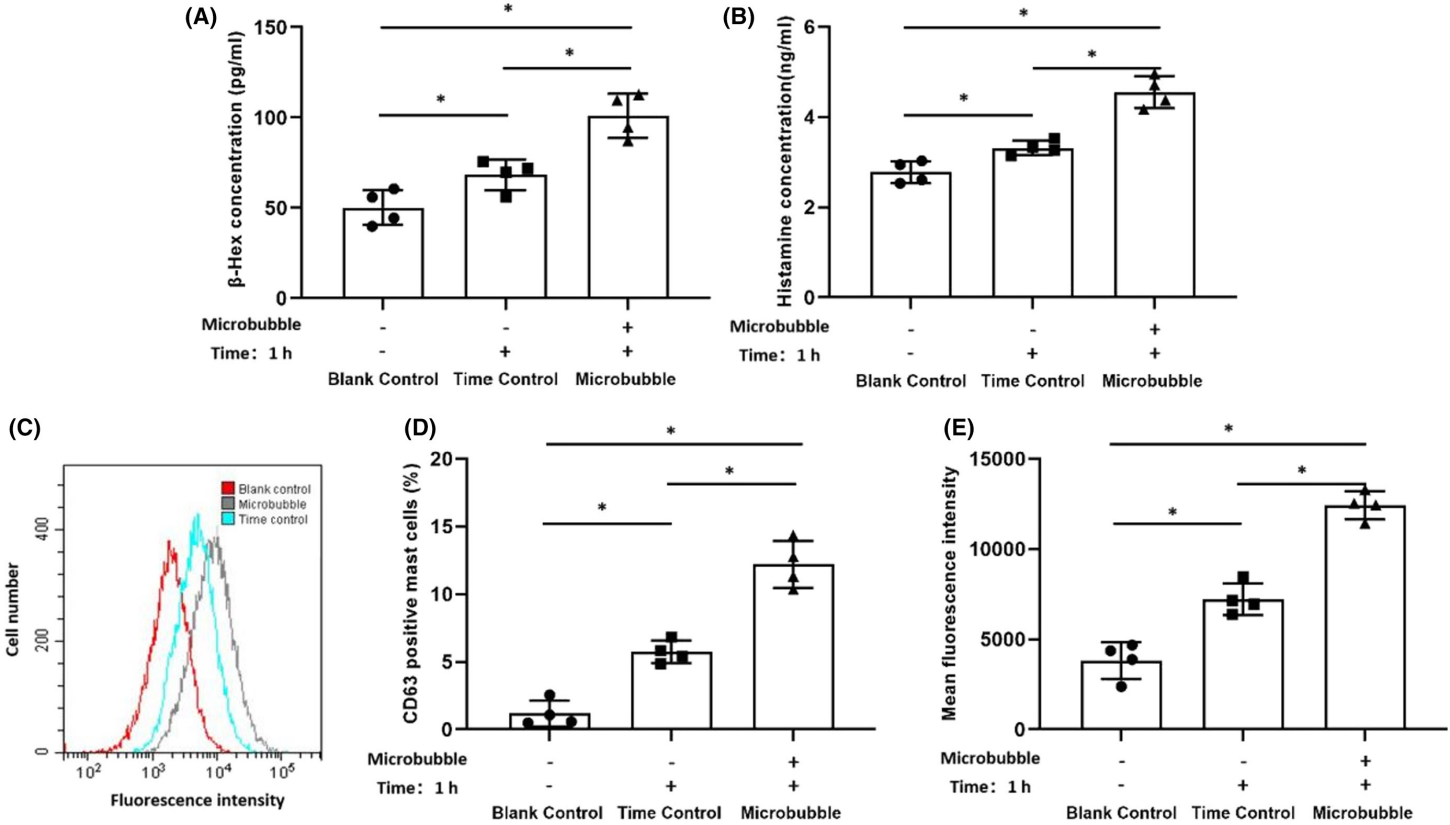
Effects of microbubbles on the degranulation and activation ability in the BMMCs. After BMMCs contacting with microbubble for 1 h, the supernatants and cell pellets were collected for analysis. Unstimulated BMMCs at different time‐points (0, 1 h) were regarded as blank and time control groups, respectively. BMMCs suspensions were centrifuged and the supernatants were used for β‐hexosaminidase and histamine detection by ELISA (A, B). BMMCs labelled by CD63 antibodies were measured by flow cytometry, and the percentage of CD63 positive mast cells and mean fluorescence intensity were compared among groups (C, D, E). Data are expressed as mean ± SD and obtained from four independent experiments for each group. *p < 0.05.

The release of histamine and β‐hexosaminidase in BMMCs supernatants were measured by ELISA. The results showed the level of β‐hexosaminidase and histamine (101.052 ± 12.209 pg/mL, 4.555 ± 0.351 ng/mL) in microbubble contact group were significantly higher than those in the blank control group (50.203 ± 9.695 pg/mL, 2.651 ± 0.451 ng/mL) and the time control group (72.863 ± 12.841 pg/mL, 3.263 ± 0.086 ng/mL). The time control group was also higher than the blank control group (F_1_ = 19.106, F_2_ = 33.897, *p* < 0.05) (Figure 2A,B).

The activated marker CD63 expression was analysed by flow cytometry. After stimulated with microbubbles, the percentage of CD63 positive cells and mean fluorescence intensity (12.195 ± 1.739%, 12423.450 ± 768.844) markedly increased compared with untreated cells in the blank control group (1.175 ± 0.960%, 3825.325 ± 1020.699) and the time control group (5.725 ± 0.829%, 7226.200 ± 874.136). The time control group was also higher than the blank control group with significant difference observed (F_1_ = 79.444, F_2_ = 93.869, *p* < 0.05) (Figure 2C,D,E).

### 3.2 Changes of intracellular calcium in BMMCs by microbubbles

**FIGURE 3:**
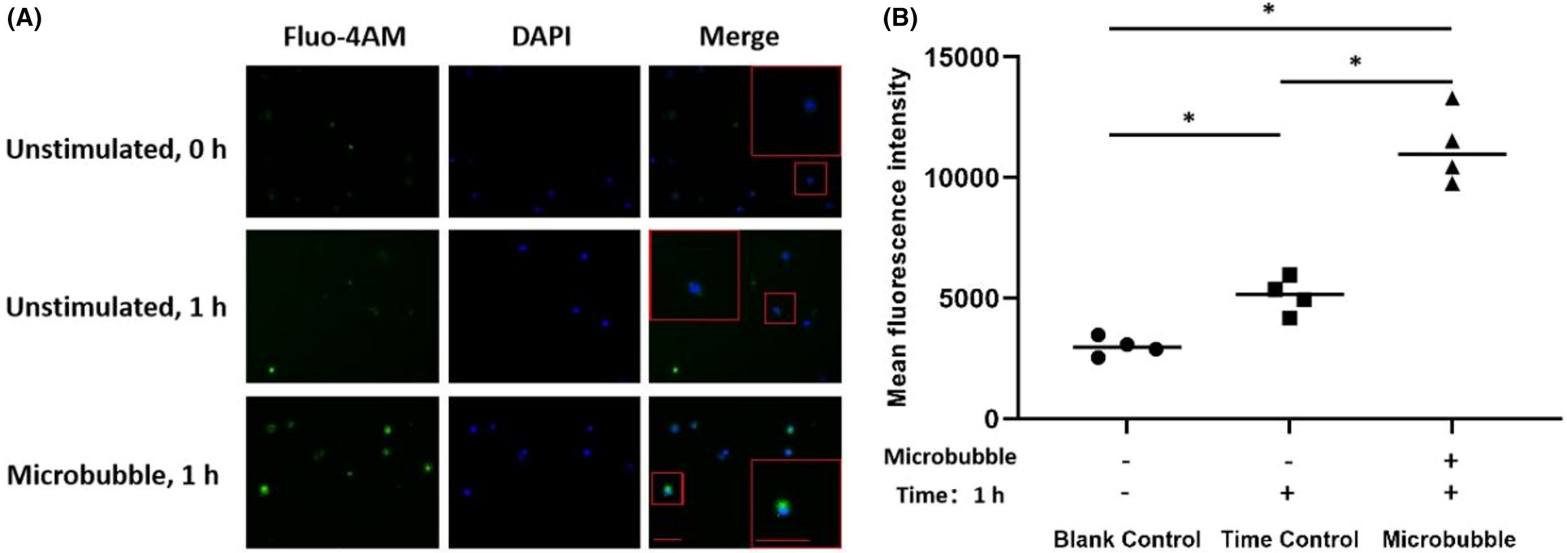
Changes of intracellular calcium in BMMCs by microbubbles. Intracellular calcium levels in BMMCs loaded by a fluorescent dye Fluo‐4 AM were taken by fluorescent microscopy, which cell nuclei marked with DAPI staining dye (A). Mean fluorescence intensity was also quantitatively analysed by flow cytometry and compared between groups (B). Scale bar = 50 μm. Data are expressed as mean ± SD and obtained from four independent experiments for each group. *p < 0.05. BMMCs, bone marrow‐derived mast cells; DAPI, destination access point identifier.

The representative images of intracellular calcium level in BMMCs were taken by fluorescent microscopy and the mean fluorescence intensity was quantitatively analysed by flow cytometry. The results demonstrated the strongly elevated calcium level was observed in the BMMCs stimulated with microbubble for 1 h but not in the unstimulated mast cells at time points of 0 and 1 h (Figure 3A). Correspondingly, the mean fluorescence intensity of calcium (11,261 ± 1539.0) markedly increased compared with untreated cells in the blank control group (2998 ± 389.1) and the time control group (5116 ± 752.7, F = 71.644, *p* < 0.05) (Figure 3B).

### 3.3 Effects of TRP inhibitors pretreatment before microbubbles on BMMCs

**FIGURE 4:**
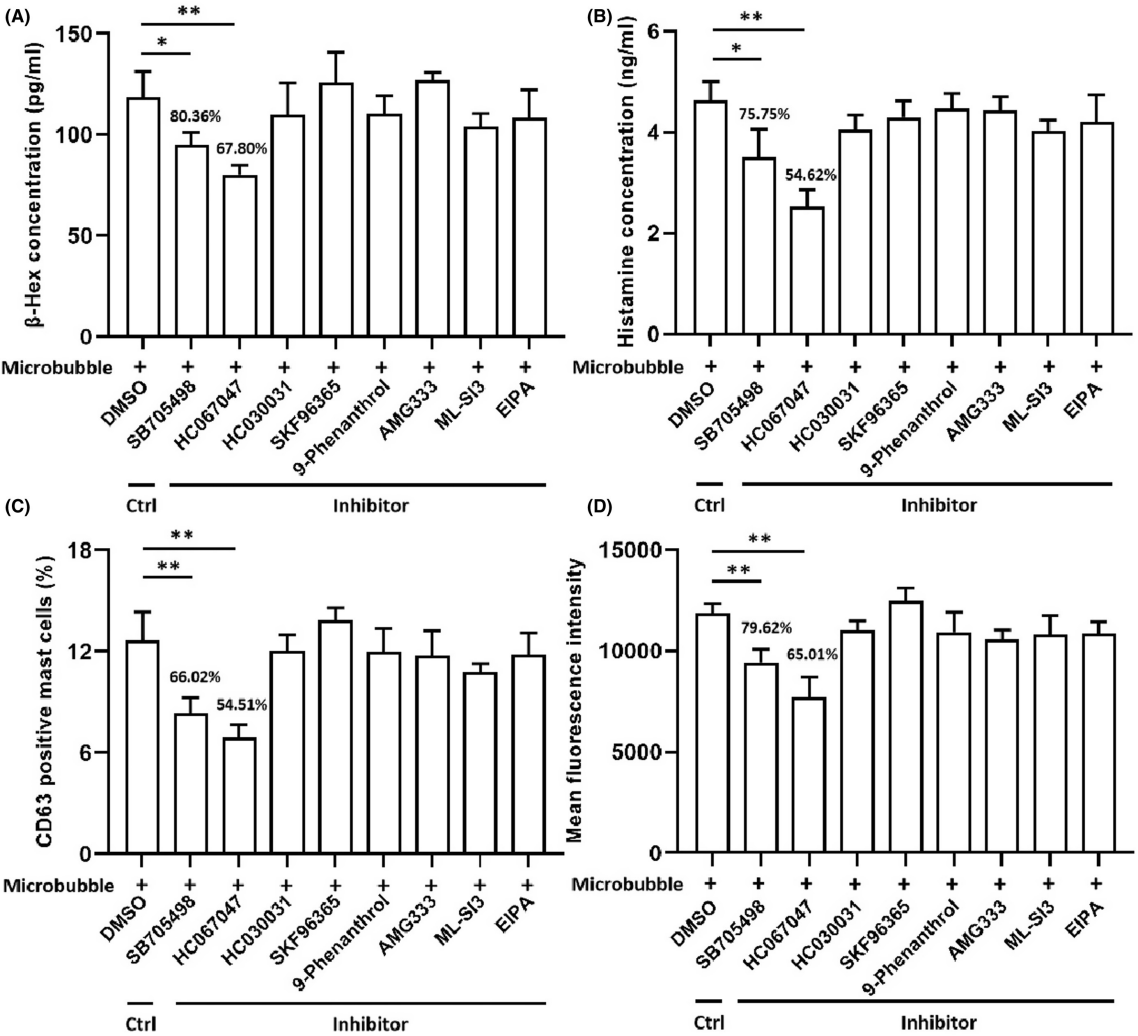
Effects of TRP inhibitors in the microbubble‐induced BMMCs. BMMCs in all groups were pretreated with different TRP inhibitors (DSMO as control) before contacting with microbubble for 1 h. The secretion of β‐hexosaminidase (A) and histamine (B), percentage of CD63 positive mast cells (C) and mean fluorescence intensity of intracellular calcium (D) were compared between multiple inhibitors pretreatment groups with control. Data are expressed as mean ± SD and obtained from four independent experiments for each group. *p < 0.05, **p < 0.01. BMMCs, bone marrow‐derived mast cells; TRP, transient receptor potential; DMSO, dimethyl sulfoxide.

Microbubbles‐evoked degranulation of β‐hexosaminidase and histamine secretion could be significantly blocked by TRPV1 and TRPV4 inhibitors, but not in other TRP subfamily channels (Figure 4A,B). Only in the groups of TRPV1 and TRPV4 inhibitors, CD63 positive mast cells percentages performed the significant inhibitory influence compared to the microbubble group (Figure 4C). Similarly, the mean fluorescence intensity of intracellular calcium tested by flow cytometry was significantly decreased via inhibiting the TRPV1 or TRPV4 channel activity. The blockage of other TRP channels including TRPA1, TRPC, TRPM4, TRPM8, TRPP3 and TRPML could not exhibit the significant changes (Figure 4D). Detailed statistical analysis results of this part are presented in Table 1.

**TABLE 1:** Results of anova and Dunnett‐*t* test.

Parameters	anova		Dunnett‐*t* test		
	F	*p*‐value	Group	Means ± SD	*p*‐value
Mean fluorescence intensity of Ca^2+^	14.854	<0.001	Microbubble	11856.901 ± 486.109	
			TRPV1	9437.691 ± 648.493	<0.001
			TRPV4	7708.195 ± 974.786	<0.001
			TRPA1	11004.723 ± 472.246	0.414
			TRPC	12457.557 ± 663.159	0.787
			TRPM4	10941.561 ± 977.247	0.342
			TRPM8	10579.092 ± 447.639	0.091
			TRPML	10816.014 ± 942.279	0.225
			TRPP3	10874.247 ± 554.059	0.275
CD63^+^ percentage	14.626	<0.001	Microbubble	12.667 ± 1.681	
			TRPV1	8.363 ± 0.906	<0.001
			TRPV4	6.905 ± 0.763	<0.001
			TRPA1	12.053 ± 0.948	0.967
			TRPC	13.838 ± 0.751	0.585
			TRPM4	11.980 ± 1.461	0.941
			TRPM8	11.783 ± 1.461	0.825
			TRPML	10.798 ± 0.494	0.145
			TRPP3	11.838 ± 1.276	0.864
β‐hexosaminidase (pg/mL)	7.578	<0.001	Microbubble	118.302 ± 12.98	
			TRPV1	95.063 ± 6.303	0.030
			TRPV4	80.203 ± 4.719	<0.001
			TRPA1	109.780 ± 16.134	0.813
			TRPC	125.941 ± 15.018	0.878
			TRPM4	110.494 ± 8.876	0.867
			TRPM8	127.069 ± 3.700	0.793
			TRPML	103.982 ± 6.590	0.318
			TRPP3	108.485 ± 13.709	0.700
Histamine release (ng/mL)	8.862	<0.001	Microbubble	4.638 ± 0.369	
			TRPV1	3.513 ± 0.544	0.007
			TRPV4	2.533 ± 0.327	<0.001
			TRPA1	4.055 ± 0.281	0.307
			TRPC	4.297 ± 0.320	0.817
			TRPM4	4.465 ± 0.310	0.994
			TRPM8	4.427 ± 0.273	0.981
			TRPML	4.032 ± 0.211	0.272
			TRPP3	4.204 ± 0.538	0.612

## 4 DISCUSSION

After rapid and substantial changes of ambient pressure, the inert gas desaturates to generate bubbles in the body, which cause a series of symptoms ranged from skin to neurological, musculoskeletal and cardiopulmonary systems. However, the occurrence of skin lesions is usually a prominent clinical sign to the severity of DCS.^12^ Currently, there are three commonly accepted pathophysiological hypotheses related to the mechanism of cutaneous DCS. The most accepted one at the moment is the autochthonous bubbles hypothesis which partly proved by our previous researches.^13^ The moving subcutaneous bubbles in the skin microcirculation have been detected by ultrasound in human cutis marmorata.^1^ Histologic observations have also revealed the existence of the circulating bubbles on the local subcutaneous tissue and the consequent congestion of the surrounding lesion areas and endothelial dysfunction.^14^, ^15^ Another study from the view of ultrastructural histopathology has discovered the vasculitis signs including perivascular neutrophil infiltrates, edema and haemorrhage of cutaneous lesions in swine DCS.^16^

It is well known that mast cells are functioned versatile cells in mediating immune allergic responses and defensing against certain parasites and venoms from pathogenic insults and protecting host from fungal, viral and bacterial infections.^17^ Other than this, mast cells could participate in tissue modelling and wound healing, modulation of angiogenesis and fibrosis, even in chronic inflammatory regulation and neoplasm formation.^18^ The activation induced by exogenous materials, endogenous substance, bioactive compounds and physical stimuli such as ultraviolet radiation, high temperature, red light laser and mechanical stress has been already acknowledged.^19^ Mast cells degranulation process encompasses exocytosis and basic outputs which produce a plethora of cytoplasmic secretory granules. These granules are rich in performed mediators and synthesized de novo biological substances that directly release in the infection sites and surrounding areas.^20^ The inflammatory reactions caused by the mediators is usually related to pathological alternation of epidermal/dermal thickness, microvasculature activity, inflammatory cell infiltration, and the clinical symptoms of skin rash, pruritus and pain.^21^, ^22^, ^23^

As the reported pathological observation and clinical manifestation of DCS skin lesion are highly consistent with the symptom and histopathology induced by the activation of mast cells. In addition, current study has reported that 1% polidocanol injectable foam used for the treatment of varicose veins may cause mast cell‐related dermatological side effects like local urticaria, which was thought to be associated with mast cells.^24^ It was highly speculated that microbubbles as a kind of mechanical stimulus could activate mast cells into some extent and positively regulate its degranulation process. To this end, this study was to model microbubbles stimulate mast cells in vitro and observe its effects.

Mast cells initially derive from pluripotent myeloid haematopoietic stem cells, and get mature after migration to specific tissues, which are widely distributed throughout the body, preferentially in proximity to epithelial surfaces and around the blood vessels or peripheral nerves.^25^ BMMCs, a widely used model of primary mast cells were utilized in our study to explore the influence of microbubble stimulation. Mast cells degranulation activity was generally assessed by the expression of CD63 and the release of β‐hexosaminidase and histamine, which were commonly used as markers to determine the level of degranulation. In the stimulation experiments, homogeneous microbubbles of 1 mm in diameter for 1 h were designed because most of the decompression bubbles remained at 0.5–1.5 mm after detachment, coalescence and expansion.^26^ In addition, pre‐experiments showed that 1 h was a relatively appropriate time because of the significant degranulation induced by microbubbles and a small proportion of spontaneous degranulation (Figure S2). After exposure to microbubbles contact for 1 h, there was a remarkable increase activation and degranulation of the mast cells according to indices of the CD63 positive percentage and mean fluorescence intensity, β‐hexosaminidase and histamine release compared to control. The increase of intracellular calcium influx of mast cells could be of vital importance for the degranulation, which was verified in canonical pathway of IgE activation. This study also observed BMMCs intracellular calcium rise and the robust release of bioactive substances upon the microbubbles stimulation.

So how did microbubbles stimulation lead to BMMCs activation? There are several studies mentioned the crucial role of calcium dependent TRP channels in the mast cells responses. For instance, the extensive mast cells degranulation triggered by rapid shift in oxygen tension could be counteracted by the addition of a TRPA1 inhibitor in human mast cells.^27^ Analgesia induced by acupuncture confirmed the involvement the mechanosensitive channels including TRPV1, TRPV2 and TRPV4.^28^ Zhang et.al, has demonstrated the participation of TRPV2 channel on the degranulation of human mast cell line HMC‐1 in response to mechanical, heat and red laser light stimulation.^29^ Suzuki et.al, discovered that loss of TRPC1‐mediated calcium influx contributes to impaired degranulation in Fyn‐deficient murine mast cells.^30^ In addition, Domocos et.al, found that cinnamaldehyde‐elicited itch sensation in humans and scratching behaviour in mice are partly dependent on the roles of TRPV1 and TRPV4.^31^ Specifically based on mouse BMMCs, inhibition of TRPV channels also inhibited calcium influx.^32^ These all highlight the crucial role of TRP channels family members to mast cells function, especially in the degranulation responses.^19^ Eight available mouse specie‐specific TRP inhibitors were utilized to explore these TRP channels and find whether they were participated in microbubble‐induced BMMCs activation. The blockade of TRPV1 or TRPV4 could inhibit microbubble‐evoked calcium influx and degranulation in BMMCs except other six TRP channels. These results suggested mast cells induced by microbubbles mainly through TRPV, of which TRPV1 and TRPV4 calcium channels were the possible channels discovered to influence calcium mobilization and degranulation process.

Based on the autochthonous bubbles hypothesis, the influence of microbubbles has also displayed on endothelial cells and neurons. For instance, intravascular bubbles stimulation on endothelial cells has been confirmed the autophagy on vascular inflammation cascades and detrimental effects of endothelium injury.^33^, ^34^ Furthermore, serum injury biomarkers induced by decompression bubbles were reflection on oxidative stress and inflammatory responses in spinal cord.^35^ Microparticles formation triggered from the vasculature by bubbles was also the main cause of vascular injury or spinal decompression illness.^33^ These all implied the miscellaneous functions of microbubbles on cells in vitro and cutaneous DCS might be associated with degranulation of microbubbles‐stimulated mast cells via modulating the activation of TRPV1 and TRPV4 channels.

Collectively, this study has firmly illustrated that stimulatory activation of mast cells by microbubbles could contribute to the enhanced degranulation activity and elevated calcium influx, which could be partially suppressed by the inhibitor of TRPV1 and TRPV4 calcium channels. Since several special inhibitors of TRP channels have not discovered yet, we cannot exclude other TRP channels play a role in the progress of mast cells activation following microbubbles stimulation. Here we have for the first time employed BMMCs, a mast cell model, upon microbubbles exposure and discovered the involvement of TRPV activation. This study is preliminary and explorative, which is a supplement and inspiration to perfect the autochthonous bubble hypothesis of DCS skin lesions. The findings introduce us a potential role of microbubbles to activate mast cells via calcium dependent pathway.

## AUTHOR CONTRIBUTIONS

**Jia Liu:** Conceptualization (equal); investigation (equal); methodology (equal); software (equal); writing – original draft (equal). **Long Qing:** Conceptualization (equal); data curation (equal); formal analysis (equal); funding acquisition (equal). **Yufei He:** Data curation (equal); formal analysis (equal); methodology (equal). **Qihui Zhu:** Data curation (equal); formal analysis (equal); methodology (equal). **Weigang Xu:** Methodology (equal); writing – review and editing (equal). **Jianhua Wu:** Supervision (equal); writing – review and editing (equal).

## CONFLICT OF INTEREST STATEMENT

The authors confirm that there are no conflicts of interest.

## Supporting information


Figure S1.

## Data Availability

The data that support the findings of this study are available from the corresponding author upon reasonable request.
