# Supplementary material for: The involvement of transient receptor potential channels in mast cell activation by microbubbles
Source: J Cell Mol Med. 2023 Sep 7;27(22):3628–36. doi: 10.1111/jcmm.17947 (PMC10660621; doi:10.1111/jcmm.17947)
Supplement: Supplementary file 1 — Figure S1. [file JCMM-27-3628-s001.docx]

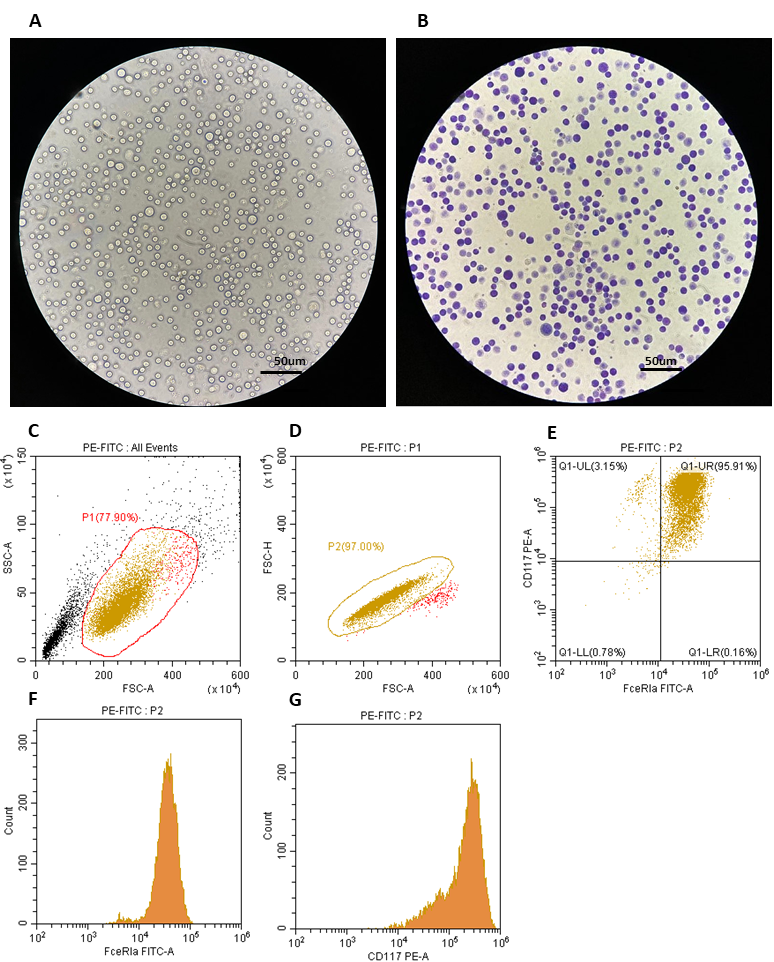


Figure S1. Culture and identification of BMMCs. Morphological characteristics of BMMCs with toluidine blue staining at 200x magnification were observed by inverted microscope (A, B). Co-expression of CD117 and FcεRⅠα on the surface of BMMCs was assessed by flow cytometry (C, D, E, F, G). Scale bar = 50um; BMMCs, bone marrow-derived mast cells.


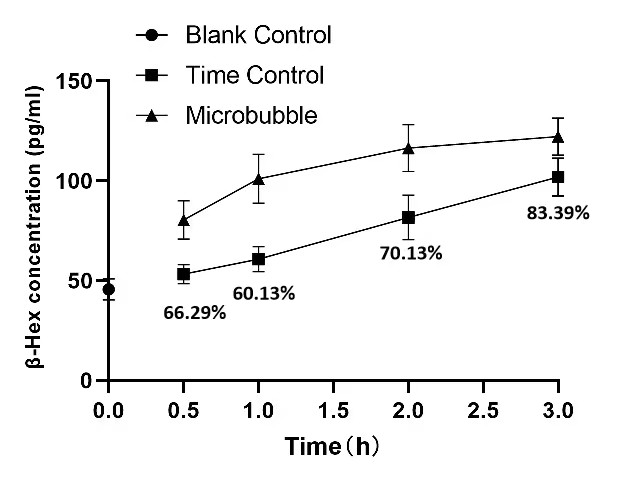


Figure S2. Trends of β-hexosaminidase release over time. The supernatants of BMMCs with microbubbles contact for 0.5, 1, 2 and 3 h were tested, and without microbubbles contact for same hours were as control group to assess spontaneous degranulation. Blank control was unstimulated BMMCs tested at the starting point to represent the baseline of β-hexosaminidase secretion. The percentages of β-hexosaminidase release level at different time points examined were marked. Data are expressed as mean ± SD and obtained from four independent experiments for each group.
